# Paradoxical Onset of Arrhythmic Waves from Depolarized Areas in Cardiac Tissue Due to Curvature-Dependent Instability

**DOI:** 10.1103/PhysRevX.8.021077

**Published:** 2018-06-26

**Authors:** Alexander S. Teplenin, Hans Dierckx, Antoine A. F. de Vries, Daniël A. Pijnappels, Alexander V. Panfilov

**Affiliations:** 1 Laboratory of Experimental Cardiology, Department of Cardiology, Heart Lung Center Leiden, Leiden University Medical Center, Leiden, the Netherlands; 2 Department of Physics and Astronomy, Ghent University, Ghent, Belgium; 3 Ural Federal University, Ekaterinburg, Russia

**Keywords:** Biological Physics, Interdisciplinary Physics, Nonlinear Dynamics

## Abstract

The generation of abnormal excitations in pathological regions of the heart is a main trigger for lethal cardiac arrhythmias. Such abnormal excitations, also called ectopic activity, often arise from areas with local tissue heterogeneity or damage accompanied by localized depolarization. Finding the conditions that lead to ectopy is important to understand the basic biophysical principles underlying arrhythmia initiation and might further refine clinical procedures. In this study, we are the first to address the question of how geometry of the abnormal region affects the onset of ectopy using a combination of experimental, *in silico*, and theoretical approaches. We paradoxically find that, for any studied geometry of the depolarized region in optogenetically modified monolayers of cardiac cells, primary ectopic excitation originates at areas of maximal curvature of the boundary, where the stimulating electrotonic currents are minimal. It contradicts the standard critical nucleation theory applied to nonlinear waves in reaction-diffusion systems, where a higher stimulus is expected to produce excitation more easily. Our *in silico* studies reveal that the nonconventional ectopic activity is caused by an oscillatory instability at the boundary of the damaged region, the occurrence of which depends on the curvature of that boundary. The onset of this instability is confirmed using the Schrödinger equation methodology proposed by Rinzel and Keener [SIAM J. Appl. Math. 43, 907 (1983)]. Overall, we show distinctively novel insight into how the geometry of a heterogeneous cardiac region determines ectopic activity, which can be used in the future to predict the conditions that can trigger cardiac arrhythmias.

## I Introduction

Arrhythmias are the most common cause of sudden cardiac death worldwide, accounting for an estimated 6 million deaths annually [1]. However, the underlying mechanisms are still not completely understood. From a practical point of view, it is of paramount importance to understand the mechanisms of arrhythmia initiation, since by removing the triggers, arrhythmic events can be avoided. Multiple recordings have shown that, in many cases, lethal cardiac arrhythmias are triggered by so-called ectopic beats, i.e., ill-timed electrical pulses originating from damaged tissue, which disturb normal cardiac rhythm [2,3]. Despite their practical importance, the mesoscopic biophysical mechanisms of ectopic activity remain largely unknown [4]. In a very general sense, ectopic activity occurs when a depolarized region in the heart produces depolarizing currents strong enough to initiate propagating waves.

The process of wave initiation by such depolarizing currents seems to be straightforward at first sight: larger currents or larger sources can more easily depolarize a cell, overcome the sink effect from adjacent cells, and produce a propagating ectopic wave.

This principle works perfectly to describe curvature-velocity relationships of waves in excitable media [5]. It closely relates to the nucleation theory of phase transitions [6,7], since one also needs to form a critical volume to initiate a phase transition in a metastable phase of matter. In electrophysiology, this paradigm is called the source-sink relationship [8].

The depolarizing current by itself may depend on many factors, including the shape of the ectopic region. The effect of the shape of injury-induced depolarized zones on ectopic activity has so far not been studied in depth because it was previously impossible to produce a depolarized region of a given shape due to various technical limitations [9,10]. Currently, this issue can be resolved by applying optogenetic techniques, which were first applied in neuroscience [11] and allow excellent spatiotemporal control of cellular properties and wave dynamics in biological excitable media [12,13]. Capitalizing on these advantages, we previously showed that optogenetically induced localized oxidative stress can be used to generate intentionally shaped zones with sustained depolarization and associated ectopic activity [14]. In the current work, this technique was applied to induce quasistable depolarized (QSD) zones of predefined shapes in monolayers of cardiac cells.

Remarkably, the ectopic activity was found to preferentially emerge from the sharp convex corners of QSD tissue regions. This observation defies the conventional interpretation of the source-sink concept, as in convex corners of the QSD region, the density of the depolarizing current is *minimal*.

In order to understand this paradoxical phenomenon, we conducted numerical simulations and reproduced the effect in physiologically detailed models and even in simple reaction-diffusion models. Below, we will link the phenomenon to the occurrence of oscillatory instability at the boundary of the QSD region and explain the onset of this instability semi-analytically using the stationary Schrödinger equation.

## II Materials and Methods

A more detailed description is provided in Appendix B.

### A Ectopic activity caused by optogenetic production of reactive oxygen species

A plasma membrane-targeted version of a mini-singlet oxygen generator (miniSOG) [15] was expressed in neonatal rat ventricular myocyte (NRVM) monolayers following lentiviral transduction, as reported previously [16,17]. Lentiviral vector particles were produced from shuttle plasmid pLV.hCMV-IE.miniSOG-PM.hHBVPRE, as detailed elsewhere [17]. The mode of action of miniSOG and the proviral DNA structure of the lentiviral vector are shown in Figs. S1(a) and S1(b) of the Supplemental Material [18], respectively. After establishing practically uniform transduction with miniSOG-encoding lentiviral vector particles, optical mapping was performed on eight- to ten-day-old cardiac monolayer cultures with the fluorescent voltage-sensitive dye di-4-ANEPPS to observe excitation waves. A patterned projection system was used to focus 470-nm LED light in the plane of the monolayers, in a region of maximal size 6 × 6 mm. After irradiation with intensity 0.31 mW/mm^2^ for 3–6 min, to induce reactive oxygen species (ROS) production, voltage was optically monitored. Cumulative ROS damage resulted in ultralong action potentials (APs) in the previously irradiated zone, lasting for 2–20 s. Light-induced ROS production did not change AP duration (APD) in the unexposed parts of the monolayers, which had an APD_80_ of 200–350 ms. The drastic spatial difference in APD caused ectopic waves to emanate from the interface between the normal and damaged tissue regions, as shown in the two examples of paired optical traces presented in Fig. S1(d) of the Supplemental Material [18]. While, in both examples, the optical signals from electrical activity in most of the irradiated region remained almost flat with a long plateau phase, the surrounding tissue exhibited periodic APs. The ectopic beats emerged only during the plateau phase of ultralong APs, in line with our previous findings [14].

### B Mathematical model for normal and QSD tissue

A hierarchy of models of different complexity was used, including the Majumder-Korhonen model for NRVMs [19], which is the model closest to our *in vitro* setup. We employed the Ten Tusscher–Noble–Noble–Panfilov (TNNP) model for adult human ventricular myocytes [20] to study *in silico* possible manifestations of the effects in human ventricular tissue. We also used the FitzHugh-Nagumo (FHN) [21] and the Aliev-Panfilov model [22], which were modified to study the generic mechanisms underlying the observed effects. A detailed description of all four models is given in Appendix B.

To identify the mechanism behind the ectopic activity observed in our *in vitro* experiments, a generic description of the tissue was used in order to relate the observed phenomena to the most fundamental properties of cardiac excitation, i.e., excitation of cardiomyocytes by local currents and establishment of a transient refractory state in the cells following excitation. In our view, this can best be achieved by employing low-dimensional models, which focus on these processes, rather than by relying on complex descriptions of the underlying ionic currents and Ca^2+^ dynamics. Simplified models often describe complex spatiotemporal phenomena without loss of essential details (see, e.g., Ref. [23]). Therefore, we used the classical cubic FHN model [21] in one (1D) or two (2D) spatial dimensions:

(1) $$\frac{\partial u}{\partial t}=-f\left(u\right)-\upsilon +D\Delta u,\;\frac{\partial \upsilon }{\partial t}=\varepsilon \left[u-\gamma \left(\vec{r}\right)\upsilon \right],$$

where *u* is the normalized transmembrane potential, *v* represents recovery processes, *f*(*u*) = *u*(*u* − 1)(*u* − *a*), 0 < *a* < 0.5, and ε ≪ 1. To distinguish between illuminated (i.e., oxidatively damaged) tissue exhibiting ultralong APs (i.e., quasistable depolarization) and normal (i.e., nonilluminated) tissue, we altered the slope of the second variable $\gamma \left(\vec{r}\right)$ as follows. Normal tissue was modeled as a monostable system (*γ* = *γ*_mono_ = 1.5), as it allows an excitable regime. After excitation (*u* ≈ 1), the system returns to the resting potential (*u* = 0); see the nullclines in Fig. 3(a). Such a monostable system supports propagating waves as well. The tissue exhibiting quasistable depolarization, however, stays in the depolarized state much longer than the duration of a normal AP. The QSD tissue reaches this depolarized state from rest only after a depolarizing wave has passed through it. The described computational model contains a slow timescale variable, which, in the limiting case, can be adiabatically eliminated [24], and QSD tissue can, therefore, be modeled as a bistable system, which can be realized in Eq. (1) by choosing *γ* = *γ_bi_* > 5.85, producing stable states *u* = 0 and *u* = *u*_3_ > 0, as shown in Fig. 3(a). The other parameters of the model were taken to be *a* = 0.13, *ε* = 0.004, and *D* = 2.0. The size of the computational domain was 1024 × 1024, with a central bistable square zone of size 400 × 400. Parameters for other shapes and models are described in Appendix B.

## III Results

### A Effect of QSD tissue geometry on ectopic beat generation in optogenetic experiments with cardiac monolayer cultures

**Fig. 1:**
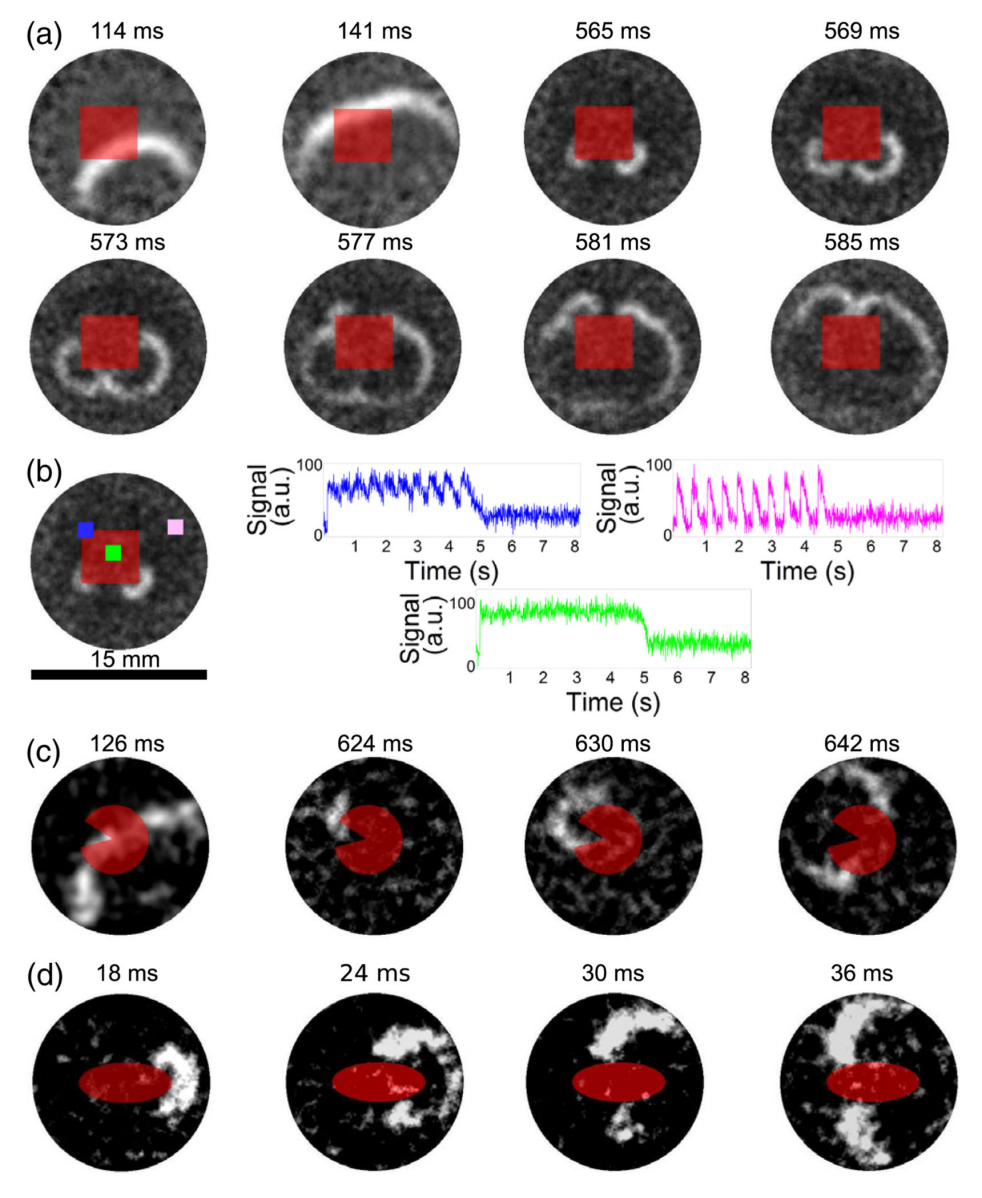
Optogenetic induction of quasistable depolarized zones (red areas) leads to ectopic waves emanating from the most curved portions of the interface between tissue with ultralong and normal action potentials. (a) Time series of a single experiment showing ectopic waves originating from the corners of a square quasistable depolarized region after a first wave has passed. (b) Distribution of the oscillations amplitude during ectopic activity at the positions indicated by the green, blue, and magenta dot. (c) Generation of an ectopic wave from the “jaw tip of a Pacman.” (d) Generation of an ectopic wave from the vertex of an elliptic interface. In all figures, the symmetry is broken by the first wave passing through the medium.

In the first set of experiments, we generated ultralong APs in a square region (i.e., QSD region) of 6 × 6 mm by local light-induced production of ROS and studied its effect on AP propagation. Following electrical stimulation, an initial wave of excitation propagated through the tissue, inducing long-lasting depolarization inside the square. This generated secondary excitation waves (i.e., ectopic beats) at the corners of the previously illuminated area, which subsequently traveled through the medium; see Fig. 1(a). The spatiotemporal organization of electrical activity during the ectopic beats is shown in Fig. 1(b). Consistent with our previous findings, the center of the illuminated area (green trace) entered into a prolonged state of depolarization (i.e., became QSD) without significant oscillations, while the unexposed tissue produced normal APs (magenta trace). The border zone between both regions showed larger oscillations (blue trace) than the center of the illuminated area. Moving from the center to the periphery of the cell monolayer, there were gradients in the amplitude of the oscillations indicative of electrotonic effects. The observed wave emission is paradoxical, for the following reason. The square region produces depolarizing electrotonic currents that excite the normal surrounding tissue of lower membrane potential. The density of the depolarizing current obviously depends on the shape of the heterogeneity, and it will be lowest at its corners, where the ratio of depolarized to repolarized tissue is minimal. Because of this unfavorable source-sink relationship, the corners of the square are the least likely regions for impulse generation, which is expected to occur in the most concave regions of the interface, e.g., in the middle of the square’s edges.

To further study how the boundary geometry affects ectopic activity, additional optogenetic experiments were performed, in which areas of ultralong APD had a Pacman-like or elliptic shape; see Figs. 1(c) and 1(d). In line with the results presented in Fig. 1(a), the first ectopic beat emerged from the “jaw tip” of the Pacman rather than from its “mouth,” as shown in frames 2–4 of Fig. 1(c). A similar result was obtained for the elliptic heterogeneity, since once again the ectopic activity was generated at the sites of highest curvature (i.e., the vertices) [Fig. 1(d)]. In total, such dynamics was observed in 29 out of 36 experiments for the square-shaped heterogeneity, in 5 out of 5 cases for the Pacman-like heterogeneity, and in 3 out of 4 cases for the elliptical heterogeneity. Our *in vitro* experiments, thus, robustly show that, contrary to what would be expected based on classical source-sink considerations, ectopic activity can originate from the parts of the depolarized region with the highest curvature, where the density of the depolarizing electrotonic current is lowest.

### B Numerical simulations of ectopic beat generation at the boundary of QSD regions

**Fig. 2:**
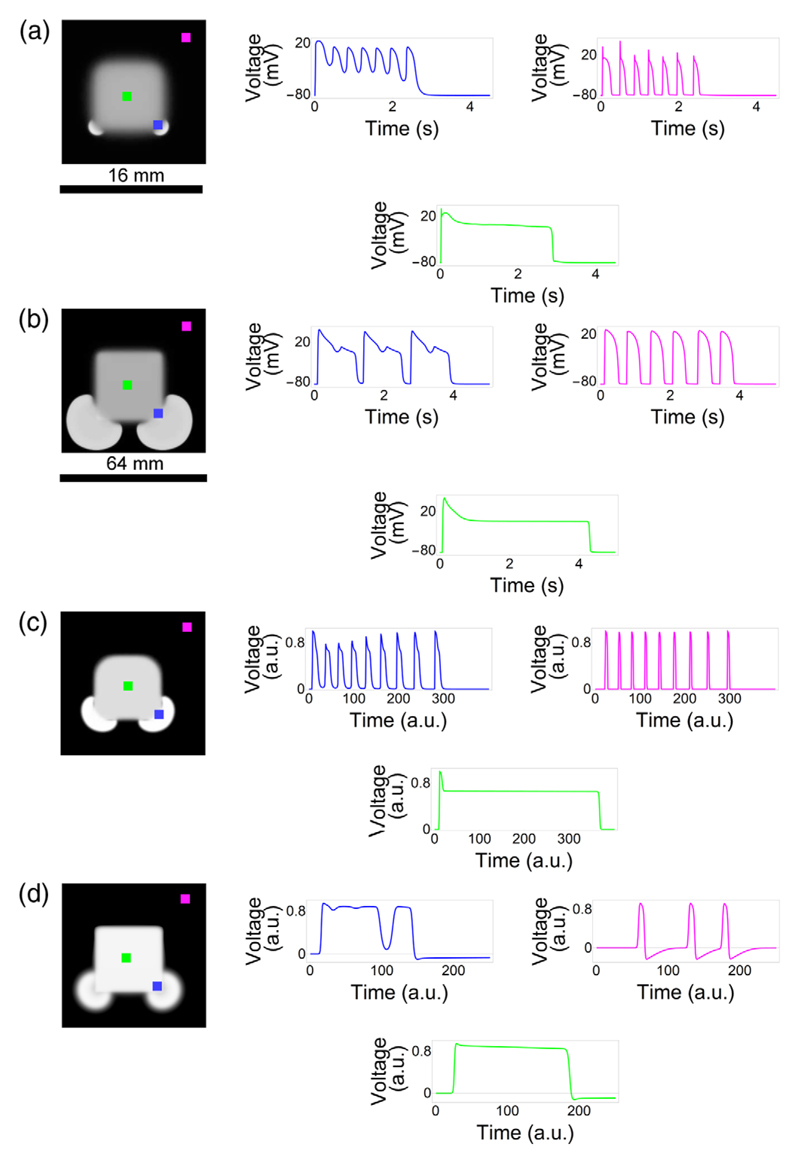
Numerical modeling of ectopic wave generation from corners of a square quasistable depolarized region in four different cardiac tissue models. For each model, a snapshot of ectopic activity and voltage signals at three different locations, indicated by the green, blue, and magenta dots, are presented. (a) Majumder-Korhonen model of neonatal rat ventricular myocytes. (b) ten Tusscher–Noble–Noble–Panfilov model of adult human ventricular myocytes. Both detailed physiological models contained an increased conductance and slow inactivation variable of the late Na^+^ current in the QSD tissue. (c) Aliev-Panfilov model. (d) FitzHugh-Nagumo model. Both of these simplified models contained a slow repolarizing variable, in addition to the bistable kinetics of the QSD tissue. In all snapshots of ectopic activity, the symmetry is broken by the first wave passing through the medium.

**Fig. 3:**
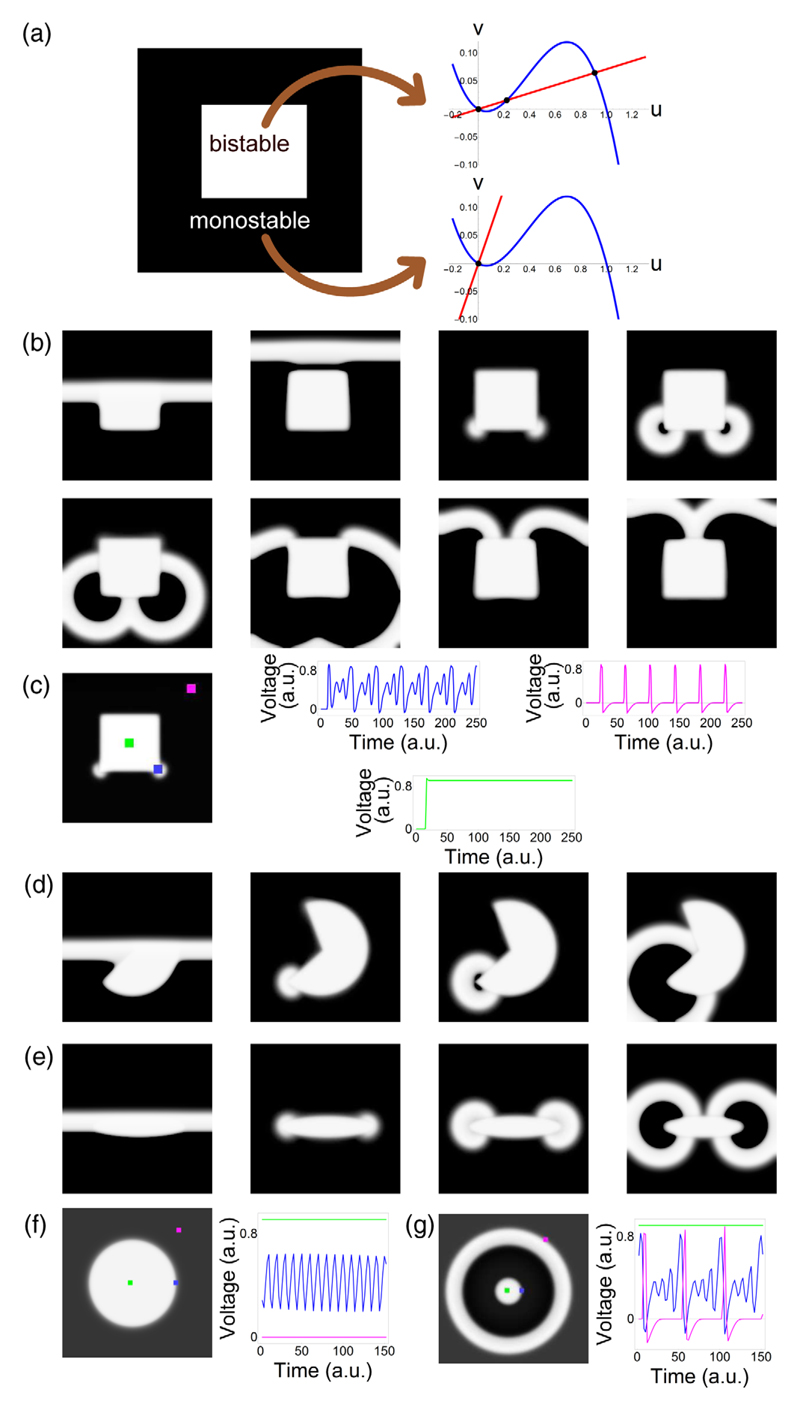
Numerical modeling of ectopic waves in the FitzHugh-Nagumo model. (a) Modeling scheme. White zones with ultralong action potentials (i.e., quasistable depolarization) are represented by a bistable system (*γ* = 14), while the surrounding black zone (normal tissue) is modeled as a monostable system (*γ* = 1.5). (b) Snapshots of ectopic beat generation from the corner of a quasistable depolarized square after passing of an initial wave [compare to Fig. 1(a)]. (c) Distribution of oscillation amplitude for oscillatory activity in (b); [compare to Fig. 1(b)]. (d),(e) Preferential generation of ectopic activity from the highest curvature areas of Pacman-shaped (d) and elliptic (e) regions [compare to Figs. 1(c) and 1(d), respectively]. The symmetry is broken by the first wave passing through the medium in (b) and (d). The wave was coming from the bottom of the domain. (f),(g) Ectopic wave potentiation by the curvature of the border zone in radially symmetric cases. Time traces, corresponding to snap-shots, are indicated by green, blue, and magenta lines. (f) Sub-threshold nonpropagating oscillatory response from a disc of large size (low curvature). (g) Propagating periodic response from a disc of small size (high curvature).

The experimental results described above were reproduced in numerical simulations of the detailed Majumder-Korhonen model of the NRVM monolayer [Fig. 2(a)]. Similar results were obtained for the detailed human TNNP ventricular model [Fig. 2(b)]. Thus, both in modeling studies and in *in vitro* experiments, the ectopic activity always originates at the corners of the QSD region. This holds true for a wide range of APDs in the QSD region; e.g., the phenomenon can be observed *in silico* even for QSD zones with moderately prolonged (1–1.2 s) APDs, resulting in the generation of just one ectopic beat from the corners (Fig. S5 of the Supplemental Material [18]). As also *in vitro* moderately prolonged APDs give rise to ectopic activity from the corners of a square QSD region (Fig. S6 of the Supplemental Material [18]), this phenomenon appears to be very robust. Although ionic models accurately reproduce the biophysical mechanism of AP generation, in a mathematical sense, they are quite complex. Thus, in order to isolate the fundamental mechanisms driving the observed phenomenon, it is always beneficial to reproduce it using a minimalistic generic description. We were able to reproduce this effect using simplified low-dimensional models, i.e., modified versions of the Aliev-Panfilov and FHN models [Figs. 2(c) and 2(d), respectively]. A detailed motivation of the simplified models choice is presented in Appendix B. As a further simplification, we were able to reproduce the same effect when the QSD region was represented as a bistable system and the normal cells as a monostable system [Fig. 3(a)], which was achieved by changing the parameter *γ*. This produced similar results as obtained *in vitro*. [Compare Figs. 1(a)–1(d) with Figs. 3(b)–3(e), respectively.] The ectopic activity originated from the corners of the bistable (i.e., QSD) region, as in the *in vitro* experiments. Similar data were obtained for the bistable/monostable version of the Aliev-Panfilov cardiac tissue model [22] (Fig. S2 of the Supplemental Material [18]). These results suggest that ectopic activity is more likely to occur in high-curvature border zones of the bistable (i.e., QSD) region than in border areas with low curvature. Consistently, a small discshaped bistable region yielded a propagating response in the surrounding tissue [Fig. 3(f)], but a larger QSD disc did not [Fig. 3(g)]. The fact that the effect persists even in a simple computer model implies that it is related to fundamental properties of excitation. Looking closer to the initiation mechanism of the ectopic waves, we found that the interface at the boundary of the bistable region exhibits oscillatory activity [Figs. 3(c), 3(f), and 3(g)]. In the simulation result Fig. 3(c), two oscillations of small amplitude are followed by one with a larger amplitude. The larger oscillation then forms an impulse propagating from the corner of the QSD region into the surrounding normal tissue. These numerical data strongly resemble the *in vitro* results of Fig. 1(b). Because of the presence of noise in the optical mapping experiments, the number of smaller amplitude oscillations needed to produce a propagating impulse in a real cardiac monolayer could not, however, be reliably determined. The importance of these oscillations was therefore scrutinized in simulations, in which the potential in the bistable region was clamped to its higher stable value *u* = *u*_3_. In this case, ectopic activity from the corners disappeared. Instead, the system generated a single pulse propagating from the entire border of the heterogeneity, which was followed by the establishment of a steady-state spatial distribution of voltage (Fig. S3 of the Supplemental Material [18]).

**Fig. 4:**
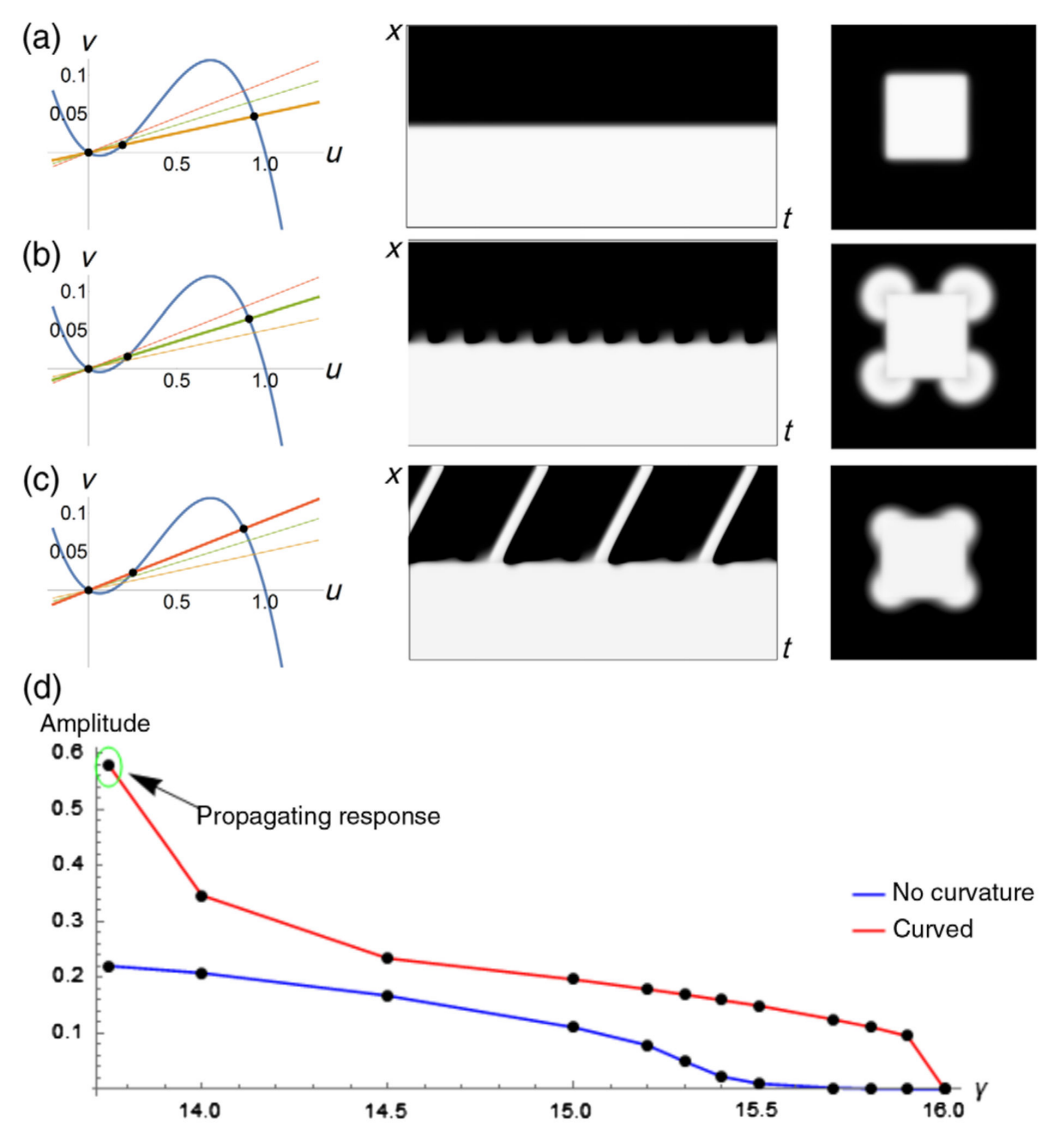
Influence of the nullcline slope 1/*γ* of the bistable zone on ectopic wave generation. (a)–(c) Induction of ectopic activity for *γ* = 20 (a), 14 (b), or 11 (c). Left panels: Nullclines. Middle panels: Space-time plots of 1D simulation. Right panels: Snap-shot from 2D simulation. (a) For *γ* = 20, only the stationary solution is found in 1D and 2D. (b) For *γ* = 14, subthreshold oscillatory activity is seen in 1D and periodic waves emanating from the corners in 2D. (c) For *γ* = 11, a propagating response is observed both in 1D and 2D. Like in (b), the ectopic origins are located in the corners of the bistable regions. (d) Bifurcation diagram for a curved (*R* = 36.0, red line) and flat (blue line) interface between the monostable (i.e., excitable) and bistable (i.e., quasistable depolarized) regions.

To further study the boundary oscillations that are responsible for ectopic beat generation, we performed simulations for different nullcline slopes *γ_bi_* in the bistable region (Fig. 4), keeping the same 2D setup as in Fig. 3(a). Numerical calculations in the 2D domain were compared to 1D simulations modeling the presence of a planar QSD region. Numerically computed stationary solutions were used as initial conditions. The following changes were observed. First, for *γ_bi_* = 20, i.e., deep in the bistable regime, no oscillations occurred at the interface [Fig. 4(a)]. For *γ_bi_* = 14 [Fig. 4(b)], oscillations emerged in 1D at the interface that did not produce any waves. However, in 2D, a periodic propagating response from the corners of the square was observed. Finally, for *γ_bi_* = 11 [Fig. 4(c)], low amplitude oscillations occurred at the interface between the mono- and bistable regions and periodically produced ectopic activity, both in 1D and in 2D, which in the latter case arose at the corners.

By plotting the amplitude of oscillation versus *γ_bi_*, the bifurcation curve for a 1D domain [Fig. 4(d), blue line] was generated. This curve showed that, at *γ_bi_* ≤ 15.5, oscillations with increasing amplitude arose. A similar bifurcation curve was generated for a circular depolarized region with radius *R* = 36.0 (red line). In this case, the bifurcation already occurs at *γ_bi_* = 15.9. Thus, we again find that curvature makes the onset of instability easier, in line with the observed ectopic wave emission from corners. However, still it is not clear why curvature shifts the bifurcation point.

## IV Mechanism of Curvature-Dependent Instability

Two different approaches were chosen to explain the curvature effect: (i) the curvature-velocity relationship and (ii) the Schrödinger equation analogy by Keener and Rinzel [25].

### A Qualitative explanation by backward motion of wavefront

First, in a very general sense, the effect of positive curvature on wave propagation is a reduction of propagation velocity, which can be explained by the fact that the density of local currents for a radially *expanding* (i.e., convex) wavefront is smaller than that for a planar wavefront. Oppositely, negative curvature, as present in a *collapsing* circular (i.e., concave) wavefront, results in a more favorable source-sink relationship and, therefore, faster wave propagation than no curvature. Let us now consider a stationary distribution of voltage for the planar and curved domain and find out how the curvature of the boundary can contribute to the onset of instability. The instability can occur either by a slight shift of the stationary solution in the forward direction (i.e., towards the normal tissue) or by a similar shift backwards. The forward motion, which is similar to the expansion of a wavefront, should generally be inhibited by curvature, while backward motion should be strengthened by it. The importance of either effect can be assessed numerically, since, in the radially symmetric case, one can write the 2D Laplacian from Eq. (1) as Δ*u* = (∂^2^*u*/∂*r*^2^) + (1/*r*)(∂*u*/∂*r*), where *r* is the radial coordinate and the term *I*_curv_ = (1/*r*)(∂*u*/∂*r*) accounts for the wavefront curvature. We performed simulations in which *I*_curv_ was present only in the normal or bistable region. We found that, when *I*_curv_ was present inside the QSD region only, it enhanced the instability. For example, in the case of a circular depolarized region with a radius *R* = 36.0, the ectopic waves started to appear for *γ_bi_* = 15.3, compared to *γ_bi_* = 13.9 in the fully radially symmetric system. However, in the opposite case, when *I*_curv_ was present outside the bistable area, the formation of ectopic activity was observed for *γ_bi_* < 12.5 only. Thus, under these circumstances, the curvature of the wavefront hindered the development of ectopic activity. Overall, we can conclude that informally the observed deviation from the conventional source-sink mismatch concept can be explained by the fact that curvature potentiates the initial backward motion of the wave to the inside of the QSD region, which breaks the stability of the system and eventually results in the onset of ectopic activity.

### B Semianalytical study of the instability using a Schrödinger equation analogy

To further analyze the oscillatory instability leading to ectopic activity, we extended the theoretical approach by Keener and Rinzel in Ref. [25]. However, in order to apply it, a simplification is introduced. The oscillatory instability occurs in the coupled mono- and bistable system and, thus, is present in both QSD and normal regions. In other words, the presence of the monostable region triggers activity in the border zone of the bistable region, leading to ectopic beat generation. However, in order to perform an analytical analysis, we need to restrict ourselves to one region only and replace the other region by a boundary condition. We keep the bistable QSD region and replace the normal (monostable) region by the Dirichlet boundary condition *u* = 0. The rationale for this is that such a boundary condition creates a current load on the QSD region similar to that of the monostable region. Indeed, if we consider a square 2D domain in a bistable regime and Dirichlet boundary condition *u* = 0 at the square’s boundary, the important features of Fig. 4 will be reproduced.

**Fig. 5:**
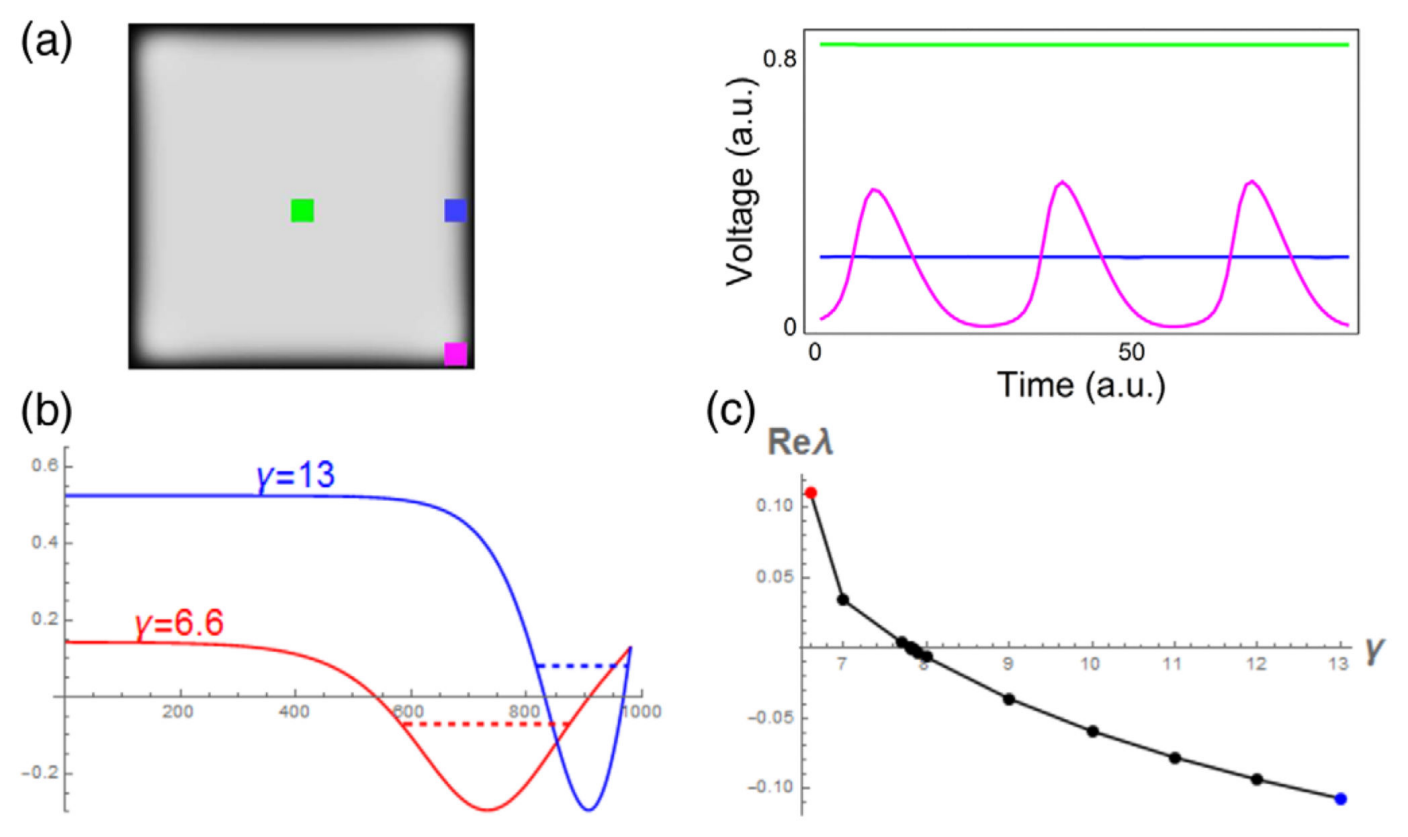
(a) Localization of oscillations in corners of a bistable domain with zero Dirichlet boundary condition and *γ* = 8.9. (b) Schrödinger potential wells with ground energy (dashed lines) values for *γ* = 6.6 (red line) and *γ* = 13 (blue line). (c) Stability parameter Re*λ* for different values of *γ*, from the ground energy calculation of the Schrödinger eigenvalue problem. The points corresponding to *γ* = 6.6 and *γ* = 13 are indicated by red and blue dots, respectively, for matching with the potential well profiles in (b).

In particular, for large values of *γ*, the solution is stable. Decreasing *γ* leads to oscillations at the boundary [Fig. 5(a)], which occur at the corners and are similar to the oscillations seen in Figs. 3(c) and 4(b). Thus, once again, we see that curvature of the boundary potentiates the instability. To account for the effects of the boundary curvature, we will perform analytical calculations for the onset of instability on an interval [*l, L*] (0 < *l* ≪ *L*) and on an annulus with radii [*l, L*] and compare the results. We choose an annulus instead of a disc because it simplifies the analysis and, for small *l*, the inner boundary has no effect on the onset of instability, which occurs at the outer boundary of the domain. Although our analysis can be extended to a disc, this will require additional estimations at *r* = 0, which would complicate presentation of the results.

Formally, we consider Eq. (1) and impose Dirichlet boundary conditions *u*(*L*) = 0 and *u*(*l*) = *u*_3_ for the 1D case and for an annulus. In both 1D and 2D, such boundary conditions allow a spatially nonuniform distribution of voltage (i.e., a smooth transition from *u* = 0 to *u* = *u*_3_), which is normally stationary in time and mimics the boundary between the QSD region and normal tissue from Fig. 3.

In this case, when *γ* is decreased, in the same way as in Fig. 4, we also observed the transition from stationary to oscillatory behavior, which is moreover facilitated by curvature.

The stability of the stationary solution in such a setup can be studied analytically. First, note that the stationary solutions (*u, v*) = [*ϕ*(*x*), *η*(*x*)] to Eq. (1) have *η* = (*ϕ*/*γ*), such that, in the absence of curvature (i.e., on a line), *ϕ*(*x*) can be found by direct integration of the following equation, which can be viewed as Newton’s equation in a potential field:

(2) $$\frac{1}{D}\left(-f\left(\phi \right)-\frac{\phi }{\gamma }\right)+\frac{\partial^{2}\phi }{\partial x^{2}}=0,\;\phi \left(l\right)=u_{3},\;\phi \left(L\right)=0,$$

where *u*_3_ is the largest root of *f*(*u*). Stability can be analyzed by linearizing around a stationary solution. Considering the perturbation *u*=*ϕ* + *ϕ*_1_, *v*=*η* + *η*_1_, with *ϕ*_1_ = *e*^*λt*^*ψ*(*x*), *η*_1_ = *e*^*λt*^*z*(*x*), yields the stationary Schrödinger equation [25]

(3) $$-\frac{\partial^{2}\psi }{\partial x^{2}}+V\left(x\right)\psi =E\psi ,$$

with potential *V*(*x*) = *f*′[*ϕ*(*x*)]/*D* and energy *E* = −[*λ*+ (*ε*/*λ* + *εγ*)]/*D*, subjected to the boundary conditions *ψ*(*l*) = 0, *ψ*(*L*) = 0. The stability of the stationary solution is lost when *λ*, which can be found from the energy *E* = −[*λ* + (*ε*/*λ* + *εγ*)]/*D*, has a positive real part [25]. From the relation between *E* and *λ*, one can see that decreasing *E* leads to destabilization of the solution, a property that will be used below. Note that, since *ϕ*(*x*) is monotonously decreasing and *f*′ is a quadratic function, *V*(*x*) has the shape of a potential well; see Fig. 5(b).

Our numerical simulations have shown that the instability occurs when *γ* is decreased below a critical value. Since a change of *γ* alters the stationary solution *ϕ*(*x*) of Eq. (1), it affects the shape of the potential well *V*(*x*), but not its depth. Representative cases are shown in Fig. 5(b) together with the numerically computed ground energy level for this potential. Lowering *γ* widens the well, which decreases the energy of the ground state and, thus, facilitates the onset of instability. Figure 5(c) shows the real part of *λ* as a function of *γ* calculated from the ground state energy in Schrödinger’s equation, predicting a critical value of *γ* = 7.813 and a nonzero imaginary part of *λ* at this value, indicating a Hopf bifurcation. We have also determined the critical value of *γ* by direct numerical calculations and found it to be *γ* = 7.810, thus very close to the theoretically predicted value. Now let us consider the effect of curvature of the domain boundary at *x* = *L* (representing the edge of the depolarized region) on the onset of the instability. In this case, stationary solutions {*ϕ_r_*(*r*), *η_r_*(*r*)} can be found from

(4) $$\frac{1}{D}\left[-f\left(\phi_{r}\right)-\frac{\phi_{r}}{\gamma }\right]+\frac{\partial^{2}\phi_{r}}{\partial r^{2}}+\frac{1}{r}\frac{\partial \phi_{r}}{\partial r}=0.$$.

The curvature term (1/*r*)(∂*ϕ_r_*/∂*r*) is equivalent to a friction force when Eq. (4) is interpreted as a particle in a Newtonian potential. Friction generally slows down the motion, and, in Appendix A, it is shown in detail that, as a result, *ϕ_r_*(*x*) ≤ *ϕ*(*x*), making the potential well broader.

When performing stability analysis on this new steady-state profile *ϕ_r_* for the radially symmetric case, we can substitute *ψ* = *r*^−1/2^*χ*, yielding

(5) $$-\frac{\partial^{2}\chi }{\partial r^{2}}+V_{r}\left(r\right)\chi =E\chi ,\;\chi \left(l\right)=0,\;\chi \left(L\right)=0,$$

with a modified potential *V_r_*(*r*) = −*f*′[*ϕ_r_*(*r*)]/*D* − 1/(4*r*^2^). The additional centrifugal term −1/(4*r*^2^) always lowers the potential well. As a result, we have shown that the spectrum of the radially symmetric problem *E_r_* will always be lower in comparison to the case without curvature. Therefore, in our model, positive curvature of the domain boundary will always potentiate the onset of an oscillatory (Hopf) instability.

Also, when the QSD region has a more complex shape, the Schrödinger equivalence still applies. In that case, the stationary solution will produce a potential well in two or three spatial dimensions that is localized near the boundary of the QSD region. This well will be broader in the regions where the profile of the stationary solution is less steep. As, in highly curved portions of the interface (e.g., at the corners), diffusion (i.e., electrotonic) effects are more pronounced, a local broadening of the well can be expected there. The Schrödinger eigenfunctions will, thus, be localized in the broader parts of the well, and these are precisely the boundary oscillations that cause the onset of instabilities. We can now understand the emergence of ectopic beats at sharp corners from a physical principle: In the corners, the transition in voltage between QSD and normal tissue will be less steep, offering more room for boundary oscillations to develop.

## V Discussion

In this study, optogenetics has been applied to investigate the onset of ectopic activity in cardiac tissue. In our setup, the ectopic activity originated from the boundary of a QSD (ultralong AP) region. Contrary to the classical principle of source-sink mismatch, the ectopic activity preferentially arose at the boundaries between the oxidatively damaged region and the normal tissue with the highest curvature. Using *in silico* models and an analytical approach, we demonstrate that the mechanism of this effect is closely related to the occurrence of oscillatory (i.e., Hopf) instability at these sites.

We demonstrated such a mechanism in detailed physiological and simplified generic models. Since we reproduced the results in different models and uncovered the general underlying biophysical mechanism, we expect that our results might be applicable to different clinical situations, in which the APD is abnormally lengthened beyond 2 s. Such situations of APD prolongation might be a result of oxidative stress [26], drug treatment, poisoning, or genetic mutations [26–29]. Compounds with strong APD-prolonging ability include Ca^2+^ channel agonists like Bay K8644; class Ia antiarrhythmics (e.g., quinidine); unintended hERG channel blockers like the antibiotic erythromycin; certain class III antiarrhythmics (e.g., E4031); and a large variety of neurotoxins (e.g., anemone toxin). APD-prolonging mutations have been found, e.g., in the genes encoding calmodulin [30], calmodulin-dependent kinase II [31], and fast Na^+^ and L-type Ca^2+^ channels [32,33]. APDs are also drastically increased during bradycardia induced by disease or rest and sleep [28,29]. The fact that APD prolongation might occur heterogeneously due to intrinsic transmural differences in repolarization kinetics, e.g., caused by increased late Na^+^ and decreased slowly delayed rectifier currents in the midmyocardial wall [28], further increases the proarrhythmic risk. However, as we showed experimentally (see Fig. S6 of the Supplemental Material [18]) and in numerical simulations (see Fig. S5 of the Supplemental Material [18]), extreme prolongation of AP is not required for ectopic wave initiation from the corners of a QSD region. It was also found for shorter APDs of 1.2 s *in vitro*, of 1 s in the Majumder-Korhonen model of NRVMs, and of 1.5 s in the TNNP model of adult human ventricular cardiomyocytes. These APDs are well within the range of those described in multiple channelopathies [32,33]. APDs of 1–2 s can be manifested in a transient fashion during pause-induced [32,34] or rhythm-acceleration-induced [35,36] APD prolongation. Both of these disturbances lead to *torsade de pointes* arrhythmias, which are widely observed clinically for different types of long QT syndrome [37]. To sum up, our results might apply to various clinical situations given the wide range of prolonged APDs for which the emission of ectopic waves from high curvature areas was manifested.

Besides by ultralong APs, ectopy from boundary areas of high curvature can also be caused by regional multifold increases in Ca^2+^ conductance. Such a local increase in Ca^2+^ conductance can result from long-chain fatty acid accumulation due to regional ischemia [38]. We illustrated this case in Fig. S7 of the Supplemental Material [18].

Another possible example is the ectopic activity arising from the ostia of pulmonary veins, which is one of the major triggers of atrial fibrillation. It was reported that patients with a common ostium of the left pulmonary veins have a higher propensity for developing atrial fibrillation [39]. This common ostium has an elliptic shape, thus containing border areas of high curvature, which may be a reason for the higher chance of developing arrhythmias.

Apart from providing a possible explanation for the emergence of particular forms of cardiac ectopy, our results may also help to refine surgical ablation procedures. Our finding that boundary curvature can play an important role in the generation of arrhythmic waves suggests that smoothening the borders between healthy and diseased myocardium may be beneficial, and that ablation of only the sharp convex corners of damaged cardiac tissue might be sufficient to prevent ectopic activity.

Usually, ectopic or focal activity is believed to be a result of abnormal automaticity caused by early or delayed afterdepolarizations [40] or so-called injury currents from damaged tissue [3]. For injury currents, it has been reported that abnormal automaticity may originate from the coupling of an excitable cell with another cell with a higher (i.e., less negative) resting membrane potential [41–43]. Despite the fact that the resting states of both cells are stable, an oscillatory (i.e., Hopf) bifurcation can emerge in such a coupled system. A similar situation exists in Figs. 1(c) and 3(c). However, in our case, the Hopf bifurcation emerges from the coupling of multiple mono- and bistable cells in a diffusive manner.

Keener and Rinzel [25] were the first to identify this Hopf instability in a FHN system for the case of a single fiber with Dirichlet or Neumann boundary conditions at one of the ends. Here, we have extended their approach to curved domains.

Similar behavior has been observed for the interface between oscillatory and excitable regions in the Belousov-Zhabotinsky reaction [44,45]. However, no local bifurcation analysis was possible for simulation of this chemical activity, since the limit cycles already had a high amplitude.

In physical systems, curvature-induced effects have been observed in nonlinear optics of solitons [46,47], shock waves in Bose-Einstein condensate [48], the appearance of additional curvature-induced magnetic force in a magnetic shell [49], the onset of superconductivity in curved domains [50,51], the appearance of prohibited states in condensed matter physics and material science [52], and curvature-induced bound states for quantum wires [53]. In all these cases, positive curvature induced unusual behavior, which cannot be observed under normal conditions in a flat geometry. The last example is most striking and stems from the fundamental result of topological trapping of a quantum particle [54]. In Ref. [54], it was shown that a quantum mechanical particle is preferentially trapped in a region of high curvature of a spatially extended Schrödinger well. The same principle applied here: The strong curvature near corners of the QSD region lowers the energy of the Schrödinger well precisely there. Overall, it is not surprising to find a quantum mechanical analogy in a reaction-diffusion context due to the long-standing mathematical similarity of the diffusion and Schrödinger equations [55]. One can just go to imaginary time and get the similar retrograde diffusion equations. Similarity between the diffusion formalism and Schrödinger equation can also be found on more general physical grounds due to the analytical continuation connection between the Wiener integral for the Brownian motion process and the Feynman path integral [56]. Therefore, the quantum mechanical analogy of localization might be applicable to more complex cases of multiple diffusing species [44] in comparison to the simple diffusion of voltage in our case. Because of the simplicity and generality of our computer model, we would expect similar effects to be found in other areas of biology, chemistry, and physics.

Albeit we reproduced the phenomenon in complex detailed models, in our research we have intentionally focused on a simplified computer model for cardiac tissue in order to relate the effect to the most fundamental properties of cardiac excitation and to be able to perform an analytical study of the dynamics. It would, however, also be of interest to investigate the role of different ionic currents in the observed effects in a more detailed mathematical model of cardiac muscle cells.

In our paper, we were able to reproduce the experimentally observed effect of ectopy from the corners in a highly relevant mathematical model for our experimental system, namely, the Majumder-Korhonen model of NRVMs and in a detailed and more clinically relevant model of adult human ventricular cardiomyocytes. Although, in all our *in silico* models, ectopy from boundary areas with high curvature occurs for a wide range of parameters (for example, for the QSD zone, as shown in Fig. 4, 11 ≤ *γ_bi_* ≤ 14 results in corner-confined ectopy), we did not study in detail all possible regimes in such a system, e.g., ectopy from corners, faces, or middles of the region, and the locations of such regimes in the parametric space. It would, hence, be interesting to investigate all possible manifestations of ectopy in a wider class of mathematical models, including detailed models for human cardiac tissue (see, e.g., O’HaraRudy [57] and Grandi [58]) and modern low-dimensional cardiac models (see, e.g., Mitchell-Schaeffer [59] and Corrado [60]) in future studies.

The extension of our work to 3D would be of importance as well, since injured cardiac ventricles typically exhibit a complex 3D structure. The effects of curvature on ectopic activity are expected to be even more pronounced in 3D systems than in our 2D preparations.

In conclusion, we have demonstrated, using complementary *in vitro* and *in silico* models, that the border zones between damaged and healthy myocardium with the highest positive curvature are the most likely areas for the onset of ectopic activity. As the electrotonic load for formation of excitation is maximal in such areas, this effect is paradoxical. Our finding, thus, adds a new potential mechanism for cardiac ectopy and represents an additional controllable degree of freedom to prevent arrhythmogenesis.

## Supplementary Material

Supplemental Material

Appendices
