# Supplementary material for: Paradoxical Onset of Arrhythmic Waves from Depolarized Areas in Cardiac Tissue Due to Curvature-Dependent Instability
Source: Phys Rev X. Author manuscript; Available in PMC 2018 Sep 10. (PMC6130777; doi:10.1103/PhysRevX.8.021077)
Supplement: Supplemental Material [file NIHMS79262-supplement-Supplemental_Material.pdf]

Supplementary Information to "Paradoxical Onset of Arrhythmic Waves from Depolarized Areas in Cardiac Tissue Due to Curvature-Dependent Instability"

Alexander S. Teplenin, Hans Dierckx, Antoine A. F. de Vries, Daniël A. Pijnappels,* and Alexander V. Panfilov†

(Dated: April 21, 2018)

* D.A.Pijnappels@lumc.nl[1]
† Alexander.Panfilov@uGent.be[1,2,3]

I. SUPPLEMENTARY FIGURES

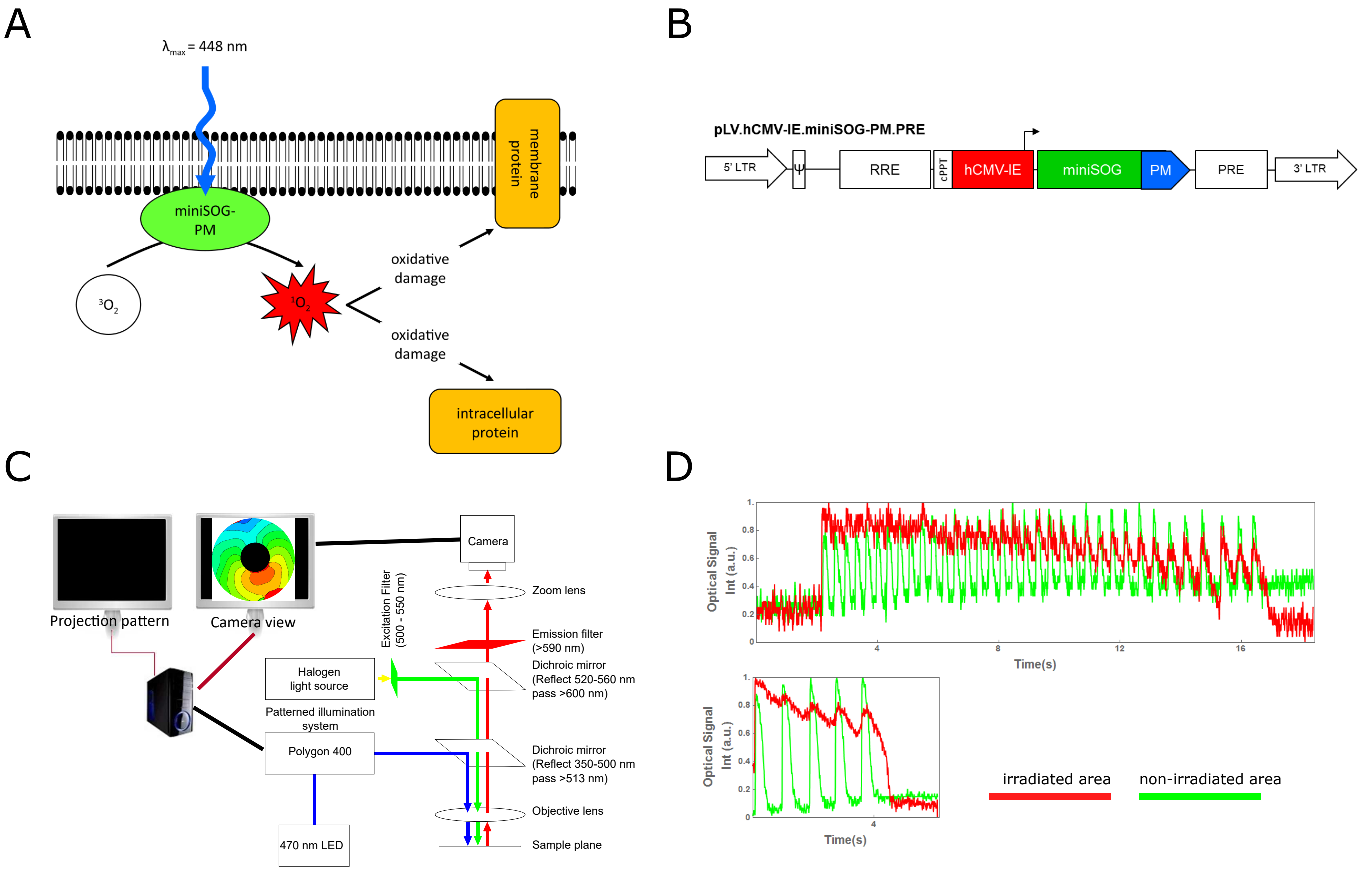

FIG. S1. Experimental setup. (*A*) Mode of action of plasma membrane-targeted mini-singlet oxygen generator (miniSOG-PM). Following excitation with blue light ($\lambda_{max} = 448$ nm), miniSOG converts molecular oxygen from its ground state ($^3O_2$) into highly reactive singlet oxygen ($^1O_2$). The oxidative damage imposed by miniSOG-PM induces a state of prolonged depolarization in neonatal rat ventricular myocytes following electrical stimulation. (*B*) Structure of the recombinant lentivirus genome in self-inactivating lentiviral shuttle plasmid pLV.hCMV-IE.miniSOG-PM.hHBVPRE. 5' LTR: chimeric 5' long terminal repeat (LTR) containing enhancer and promoter elements of the Rous sarcoma virus LTR and the human immunodeficiency virus type 1 (HIV1) R and U5 regions. Ψ: HIV1 packaging signal. RRE: HIV1 Rev-responsive element. cPPT: HIV1 central polypurine tract and termination site. hCMV-IE: human cytomegalovirus immediate-early gene promoter. hHBVVPRE: human hepatitis B virus post-transcriptional regulatory element. 3' LTR: 3' HIV1 LTR with a large deletion in the U3 region to render the corresponding lentiviral vector self-inactivating. (*C*) Setup of the optical mapping system with conjugated targeted illumination system. (*D*) Optical voltage traces of the induction of quasi-stable depolarized states characterized by ultralong action potentials in oxidatively damaged areas of confluent neonatal rat ventricular myocyte cultures.

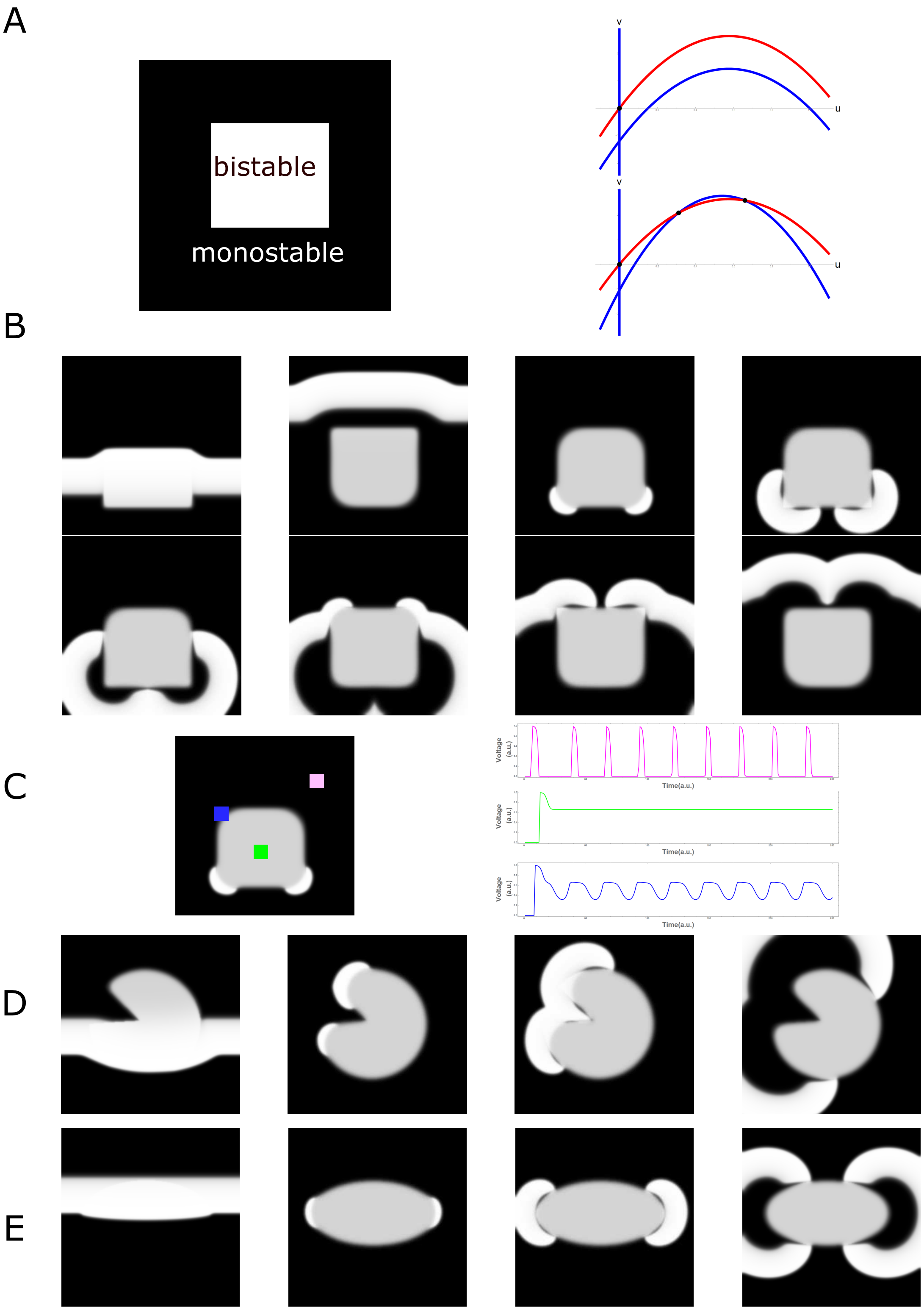

FIG. S2. Numerical modeling of ectopic waves in the Aliev-Panfilov model. (*A*) Modeling scheme. Zones with ultralong action potentials (white) are represented by a bistable system, while normal tissue (black) is modeled as a monostable system. (*B*) Snapshots of ectopic beat generation from the corners of a quasi-stable depolarized square after passing of an initial wave from the bottom of the domain. (*C*) Distribution of the oscillation amplitude in (*B*) during oscillatory activity at the positions indicated by the green, blue and magenta dot. (*D* and *E*) Local generation of ectopic activity from pacman-shaped (*D*) and elliptic (*E*) quasi-stable depolarized regions. In both cases, the ectopic activity emerges from the interfaces of the highest curvature between the mono- and bistable regions.

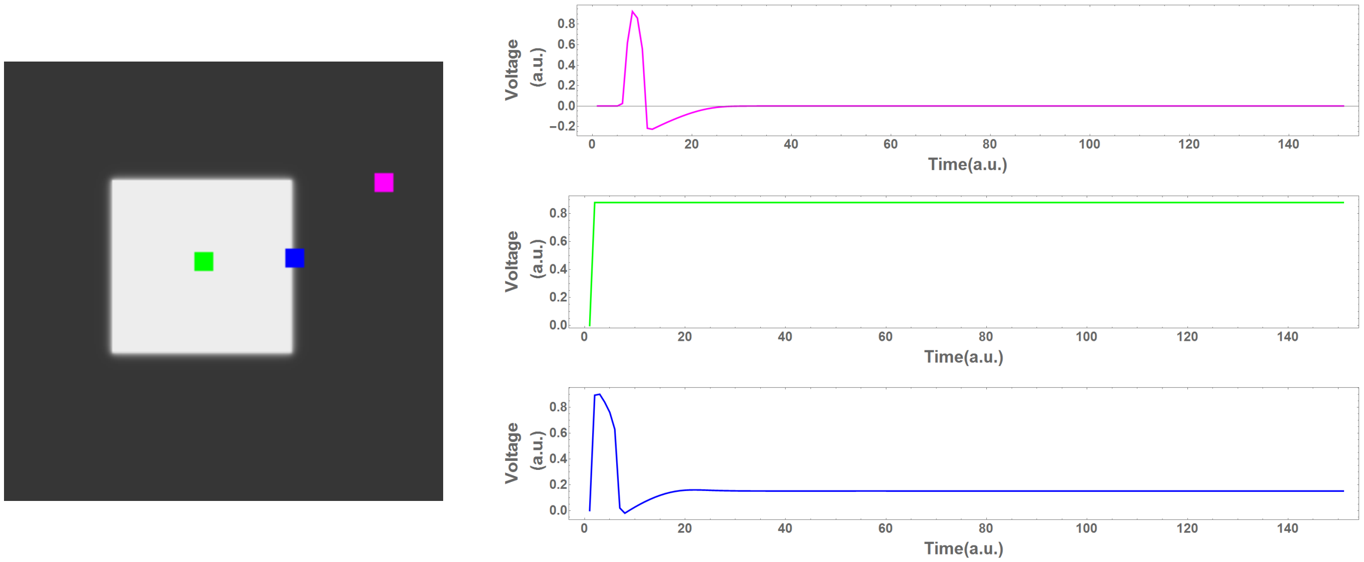

FIG. S3. Application of constant voltage does not result in oscillatory activity. The constant voltage value corresponds to the biggest stationary point on the FitzHugh-Nagumo nullcline for $\gamma = 11$.

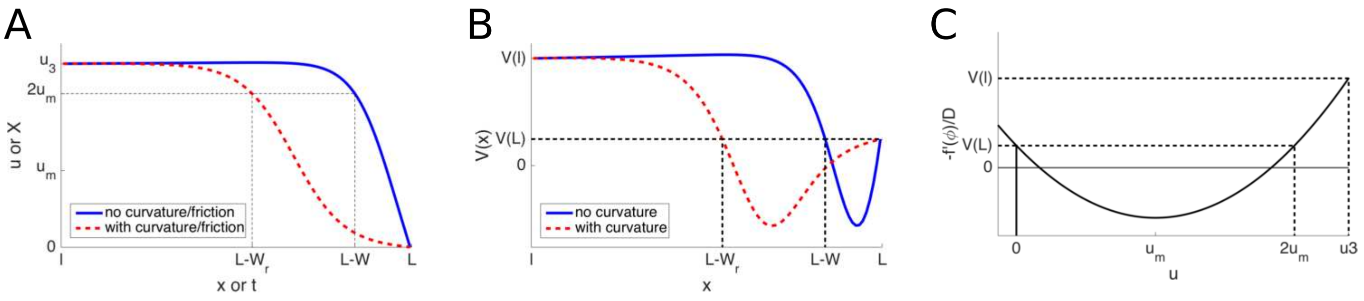

FIG. S4. Qualitative comparison of the stationary state solutions and potential wells in the cases with and without curvature effect. (*A*) Steady-state profiles. (*B*) Potential wells. (*C*) Potential function.

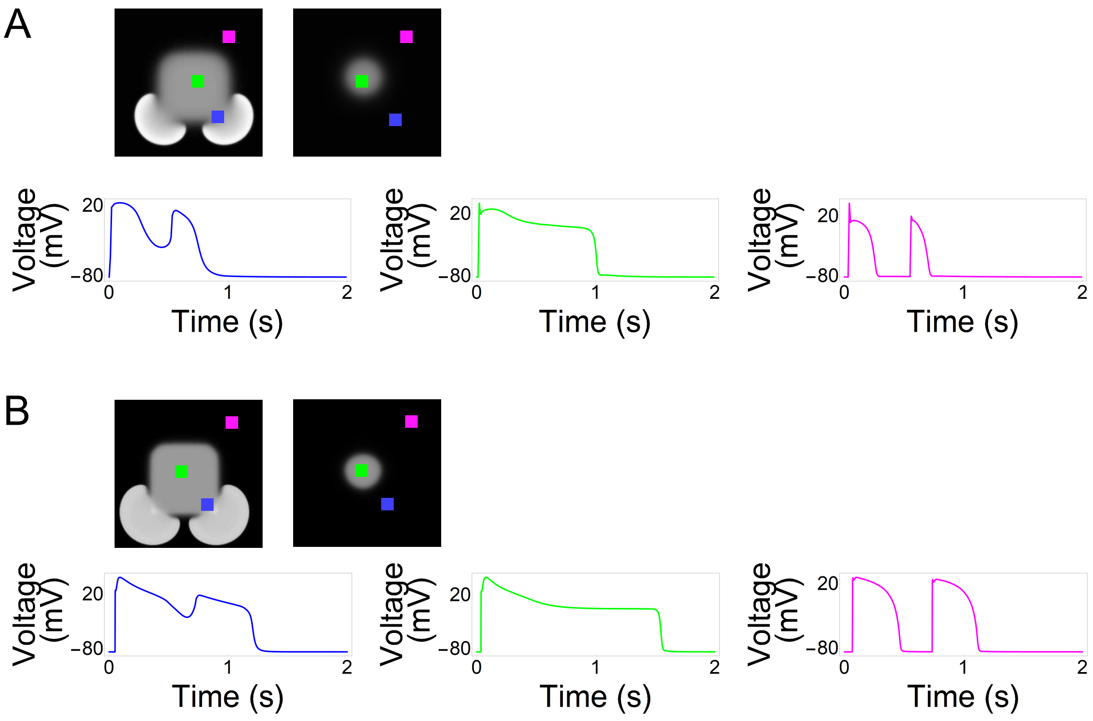

FIG. S5. Short episodes of ectopic activity (i.e. one ectopic beat) in physiologically detailed computational models. Each panel contains snapshots of two events: ectopic beat generation from the corners of a depolarized square (left panels) and nearly complete repolarization (dissipation of voltage gradients). (*A*) Majumder-Korhonen model of neonatal rat ventricular cardiomyocytes. (*B*) ten Tusscher-Noble-Noble-Panfilov model of adult human ventricular myocytes. The line graphs below the snapshots show the time traces of voltage at the positions indicated by the green, blue and magenta spots.

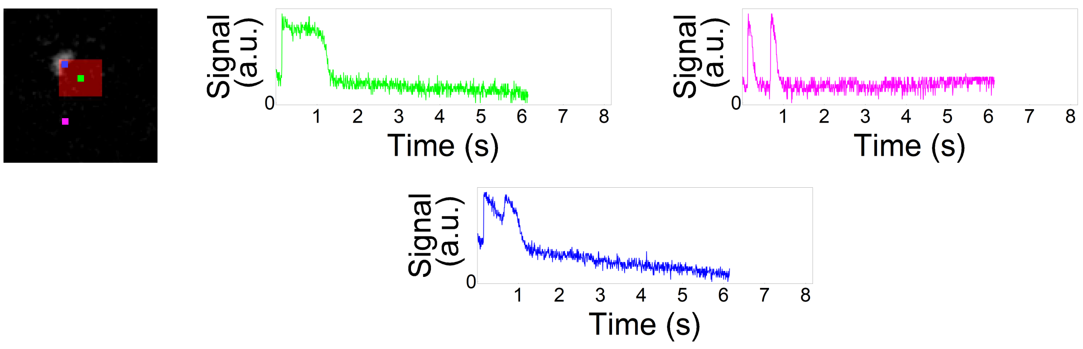

FIG. S6. Short episodes of ectopic activity (i.e. one ectopic beat) in *in-vitro* experiments. The line graphs below the snapshots show the time traces of voltage at the positions indicated by the green, blue and magenta spots.

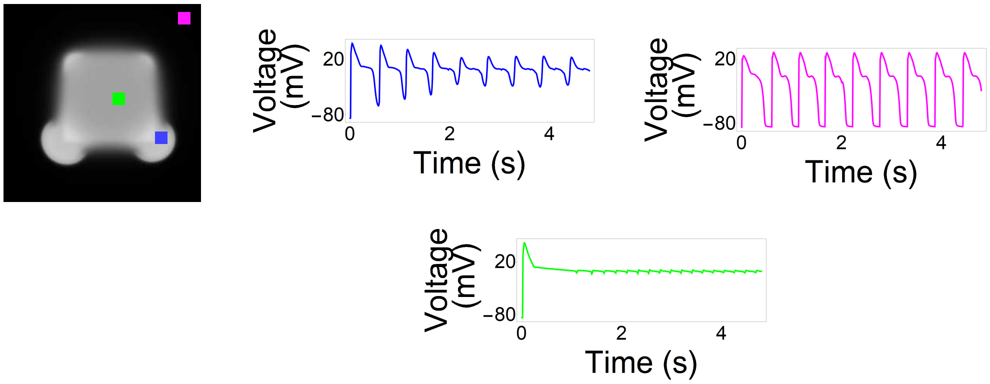

FIG. S7. Ectopic activity emerging from the corners of a square with a 6-fold higher Ca^{2+} conductance than its surroundings using the ten Tusscher-Noble-Noble-Panfilov model of adult human ventricular cardiomyocytes. The line graphs below the snapshot shown the time traces of voltage at the positions indicated by the green, blue and magenta spots.
