# Supplementary material for: Paradoxical Onset of Arrhythmic Waves from Depolarized Areas in Cardiac Tissue Due to Curvature-Dependent Instability
Source: Phys Rev X. Author manuscript; Available in PMC 2018 Sep 10. (PMC6130777; doi:10.1103/PhysRevX.8.021077)
Supplement: Appendices [file NIHMS79262-supplement-Appendices.pdf]

APPENDIX A: ANALYTICAL PROOF THAT CURVATURE PROMOTES INSTABILITY

Our proof will be based on the Schrödinger analogy first given by Rinzel and Keener. They stated that the relation $E(\lambda)$ can be inverted by solving the quadratic equation

$$\lambda^2 + (\epsilon\gamma + DE)\lambda + \epsilon(1 + \gamma DE) = 0. \qquad \text{(A1)}$$

From studying the possible locations of roots λ_1, λ_2 in the complex plane, it follows that at least one root has a positive real part if and only if $\lambda_1 + \lambda_2 = -(\epsilon\gamma + DE)$ is positive. Therefore, the stationary solution will become unstable when $E < E_0 = -\epsilon\gamma/D$. At this critical value, $\lambda_1\lambda_2 = (1 + \gamma DE_0) = 1 - \gamma^2\epsilon > 0$ in our working regime. This indicates that, at the instability threshold, λ_1, λ_2 are purely imaginary and, therefore, the system undergoes a Hopf bifurcation.

We now proceed to show that positive curvature of the boundary promotes instability of the system by proving that positive curvature of the boundary always lowers E.

Adding curvature to the system changes both the stationary profile and the associated linearized equation. We show this in two steps. First, we write the systems [1] and [3] of the main text as a particle with unit mass in a Newtonian potential: letting u correspond with 1D particle position X and x with time $t \in [l, L]$ yields

$$\frac{d^2X}{dt^2} + b(t)\frac{dX}{dt} = -\frac{dU(X)}{dX}, \tag{A2}$$

with potential $U(X) = \int^X f(\phi)d\phi$. For the case without curvature, $b(t) = 0$, corresponding to a frictionless Newtonian system; the case with curvature is equivalent to the particle being subjected to a frictional force $b(t) = 1/t$. In both cases, the boundary conditions are $X(l) = u_3$ and $X(L) = u_1$. A representative solution for both cases is given in Fig. S4(a) of the Supplemental Material [18]. Note that, since both particles need to travel the same distance $u_3 - u_1$ in the same time interval $L - l$, the frictionless case starts with a smaller initial velocity and, therefore, always lags behind the case with friction, only catching up at time $t = L$. Since $u_3 > u_1$ and the Newtonian paths were called $\phi(x)$ and $\phi_r(x)$ in the main text, we thus have

$$\phi(x) > \phi_r(x), \qquad l < x < L. \tag{A3}$$

With this result, we can compare the eigenvalues E of the linear system,

$$-\frac{\partial^2\psi}{\partial x^2} + V(x)\psi = E\psi, \tag{A4}$$

for potentials $V(x) = -f'(\phi)/D$ or $V_r(x) = -f'(\phi_r)/D$, yielding ground state eigenvalues E and E_1, respectively. Note that the term $(1/x)(d\psi/dx)$ is not included here yet; it will be performed below. A well-known property of the Schrödinger problem is that a wider and deeper well lowers the energy. For the two cases considered, the well depth is equal to $-f'(u_m)/D$, with u_m representing the unique inclination point of $f(u)$. Thus, the difference in E will only depend on the width of the potential well. To define the well width W_r or W for the cases with and without curvature, we consider the typical wells for our system as shown in Figs. 5(b) and in S4(b) of the Supplemental Material [18]. Note that $f'(\phi)$ is a quadratic function centered at $\phi = u_m < (u_1 + u_3)/2$; see Fig. S4(c) of the Supplemental Material [18]. Hence, $V(l) > V(L)$; i.e., the potential is always higher on the left-hand side, where the boundary condition is $u(l) = u_3$. Therefore, it makes sense to define the width of the potential as the region where $V(x) < V(L)$; see Fig. S4(a) of the Supplemental Material [18]. Since $f'(u)$ is a quadratic function and $u_1 = 0$, it follows that $f'(2u_m) = V(L)$, such that $W = L - \phi^{-1}(2u_m)$, $W_r = L - \phi_r^{-1}(2u_m)$. Hence, it follows from Eq. (A3) that

$$W_r > W \Rightarrow E_1 < E. \tag{A5}$$

We still have to consider the effect of adding the curvature term to Eq. (A4). However, in the main text, it is shown that a substitution $\psi = r^{-1/2}\chi$ produces again the Schrödinger problem with a potential shifted down by $1/(4r^2)$ and eigenvalue E_r. Therefore,

$$E_r < E_1 < E. \tag{A6}$$

Hence, we have established that positive curvature at the boundary of a bistable domain promotes instability. As we have only used qualitative properties of the solution, e.g., monotonicity of the profile and the asymmetric sigmoidal shape of $f(u)$, our analysis is not restricted to the FHN model. Our analysis can be extended for a disc. This would, however, require additional estimations at $r = 0$ that would unnecessarily complicate the presentation of the results.

APPENDIX B: SUPPLEMENTARY MATERIALS AND METHODS

1. Cell isolation and culture

NRVMs were isolated and cultured as reported previously [16]. In brief, hearts were excised from neonatal rats under anaesthesia, and venticular tissue was delicately chopped and dissociated with 450 U/ml collagenase type I (Worthington, Lakewood, New Jersey) and 18.75 Kunitz/ml DNase I (Sigma-Aldrich, St. Louis, Missouri). Cells were seeded on round, fibronectin-coated (Sigma-Aldrich) 15-mm-diameter glass coverslips in 24-well culture plates (Corning Life Sciences, Corning, New York). Cells were seeded at a density of 8×10^5 cells/well and incubated for 2 h with mitomycin-C (10 μg/ml, Sigma-Aldrich) to inhibit proliferation of nonmyocytes.

2. Molecular cloning

The self-inactivating lentiviral shuttle plasmid pLV.hCMV-IE.miniSOG-PM.hHBVPRE, which codes for a plasma-membrane-associated version of miniSOG, was engineered by a two-step operation. In step 1, the miniSOG-coding sequence was extended with the plasma-membrane targeting motif of human K-Ras4B by substituting the BglII × EcoRI fragment of plasmid miniSOG-C1 [15] with a linker molecule composed of oligonucleotides 5' GATC-CAAGATGAGCAAAGACGGCAAAAAGAAGAAAAA-GAAGTCCAAGACAAAGTGCGTGATCATGTAAAG 3' and 5' AATTCTTTACATGATCACGCACTTTGTCTTGG-ACTCTTTTTCTTCTTTTTGCCGTCTTTGCTCATCTTG 3' (Sigma-Aldrich). The resulting plasmid was designated

pminiSOG-PM. In step 2, the 414-bp Eco47III × EcoRI fragment of pminiSOG-PM was blunt-ended with Klenow polymerase and inserted in between the SmaI site and filled-in BsrGI site of pLV.hCMV-IE.IRES.eGFP.hHVBPRE [16]. The resulting construct was designated pLV.hCMV-IE.miniSOG-PM.hHBVPRE. The restriction enzymes and other DNA-modifying enzymes were purchased from New England Biolabs (Bioké, Leiden, the Netherlands) or Thermo Fisher Scientific (Landsmeer, the Netherlands). Large-scale plasmid isolation was performed with the JETSTAR 2.0 Plasmid Maxiprep kit (Genomed, Löhne, Germany), in accordance with the instructions supplied with the kit.

3. Lentiviral vector particle production and transduction of confluent monolayers of NRVMs

Construct pLV.hCMV-IE.miniSOG-PM.hHBVPRE was used for the production of miniSOG-encoding lentiviral vector particles employing a second-generation packaging system as previously detailed [17]. These particles were subsequently used to transduce 5-day-old continuous monolayer cultures of NRVMs with a vector dose that resulted in transduction of approximately 95% of the cells. Assessment of the transduction efficiency was done with an inverted phase-contrast and fluorescence microscope (Axiovert 35, Carl Zeiss, Sliedrecht, the Netherlands) by visualization of the green fluorescent signals produced by excited miniSOG molecules.

4. Optical mapping and patterned illumination of monolayers

After 8–10 days of culturing, ventricular monolayers were optically mapped using the voltage-sensitive dye di-4-ANEPPS (Thermo Fisher Scientific) as reported previously [17]. The mapping setup was based on a 100 × 100 pixel CMOS Ultima-L camera (Scimedia, Costa Mesa, California). The field of view was 16 × 16 mm, resulting in a spatial resolution of 160 μm/pixel. For targeted illumination of monolayers, the setup was optically conjugated to a digitally controlled micromirror device (DMD), the Polygon 400 (Mightex Systems, Toronto, Ontario), with a high-power blue (470-nm) LED (BLS-LCS-0470-50-22-H, Mightex Systems). Before starting the actual experiments, all monolayers were mapped during 1-Hz electrical point stimulation to check baseline conditions. Electrical stimulation was performed by applying 10-ms-long rectangular electrical pulses with an amplitude of 8 V to a bipolar platinum electrode with a spacing of 1.5 mm between the anode and cathode. Only cultures with an APD at 80% repolarization (APD_{80}) below 350 ms and a conduction velocity above 18 cm/s were used for further experiments. Next, monolayers were illuminated with different light patterns at constant intensity (0.3125 mW/mm^2) in the sample plane for 3–6 min. The resulting electrical activity was recorded for 6–24 s at exposure times of 1 or 6 ms per frame.

5. Data analysis

Data analysis was performed using specialized BV Ana software (Scimedia), ImageJ Ref. [61], and custom-written scripts in Wolfram Mathematica (Wolfram Research, Hanborough, Oxfordshire, United Kingdom). APD and conduction velocity were calculated as described previously [17]. To prepare representative frames of wave propagation, optical mapping videos were filtered with a spatial averaging filter (3 × 3 stencil) and a derivative filter.

6. Numerical modeling and calculations

a. Numerical calculations with simplified models

For 2D and 1D simulations, we used the forward Euler method with time step $\Delta t = 0.002$ and a centered finite-differencing scheme to discretize the Laplacian with space step $\Delta x = 0.25$. A 1024 × 1024 grid was used for the 2D simulation and a 1024-point cable for the 1D simulation. In all simplified models, spatially uniform zero initial conditions were used for all variables. To eliminate possible spatial discretization errors, we used time step $\Delta t = 0.00\,015\,625$ and space step $\Delta x = 0.03\,125$, and we used 8192 grid points for the 1D bistable system and for the Schrödinger eigenvalue problem. The Schrödinger eigenvalue problem was solved by calculating a finite-dimensional matrix eigenvalue problem using a centered second-order finite-differencing scheme for the Laplacian operator.

b. Aliev-Panfilov model

We used the modified Aliev-Panfilov model [22] to create bistable and monostable zones as shown in Fig. S2 of the Supplemental Material [18],

$$\frac{\partial u}{\partial t} = -k(u-1)(u-a) - ruv + D\Delta u,$$
$$\frac{\partial v}{\partial t} = 0.9\left(c + \left(\frac{d_1 v}{d_2 + u}\right)\right)[-v - ku(u-b-1)], \qquad \text{(B1)}$$

where $c = 0.002$, $b = 0.15$, $d_1 = 0.2$, $d_2 = 0.3$, $k = 8$, and $D = 2.0$. These parameters are the same for the bi- and monostable zone. The equations for the monostable zone were converted to bistable ones by using the threshold $a = 0.08$ and a repolarizing force $r = 0.61$. The values for the monostable zone were $a = 0.15$ and $r = 1$ as in the original Aliev-Panfilov formulation. The corresponding nullclines are shown in Fig. S2(a) of the Supplemental Material [18].

c. Implementation of ultralong APs in the FHN model

To mimic ultralong APs in a simplified model, we modified the FHN reaction-diffusion model as follows:

$$\frac{\partial u}{\partial t} = -f(u) - v - g + D\Delta u,$$
$$\frac{\partial v}{\partial t} = \varepsilon[u - \gamma(\vec{r})v],$$
$$\frac{\partial g}{\partial t} = \varepsilon_2(u - \gamma_2 g). \quad \text{(B2)}$$

Here, $f(u) = u(u-1)(u-a)$, $a = 0.13$, $\varepsilon = 0.004$, and $D = 2.0$. The state variable g was added to the standard model, having the slowest timescale $\varepsilon_2 = 0.00005 \ll \varepsilon \ll 1$. Another parameter $\gamma_2 = 3.12$. For $g = 0$, the differential equations for u and v constitute a monostable or bistable system. Normal tissue was modeled as a monostable system ($\gamma = \gamma_{\text{mono}} = 1.5$), if $g = 0$. QSD tissue was modeled as a bistable system ($\gamma = \gamma_{bi} = 14.0$), if $g = 0$.

d. Implementation of ultralong APs in the Aliev-Panfilov model

Similarly to the previous paragraph, we introduced a very slowly recovering variable g in the Aliev-Panfilov model:

$$\frac{\partial u}{\partial t} = -k(u-1)(u-a) - ru(v+g) + D\Delta u,$$
$$\frac{\partial v}{\partial t} = 0.9\left(c + \left(\frac{d_1 v}{d_2 + u}\right)\right)[-v - ku(u-b-1)],$$
$$\frac{\partial g}{\partial t} = \varepsilon_2(u - \gamma_2 g), \quad \text{(B3)}$$

where $\varepsilon_2 = 0.0001$, $\gamma_2 = 3.12$.

e. Detailed electrophysiological model of NRVMs with regionally activated late Na^+ current

The monolayer model of NRVMs was adopted from [19]. The steady-state voltage dependence for the inactivation variable h of fast Na^+ current was changed to $h_\infty = [1 + e^{[(65+V)/6.07]}]^{-1}$, where V is the transmembrane potential. In the square QSD zone, the late Na^+ current was formulated as an additional low-conductance Na^+ current, in accordance with [62]

$$I_{\text{Na}} = G_{\text{NaL}} {m_L}^3 h_L (V - E_{\text{Na}}), \quad \text{(B4)}$$

where $G_{\text{NaL}} = 1.2288\ \text{mS}/\mu\text{F}$. The formulation of the activation variable m_L coincides with the formulation of m for the fast Na^+ current and, for h_L, it is given by

$$\frac{dh_L}{dt} = \frac{h_L - h_{L,\infty}}{\tau_{hL}}. \quad \text{(B5)}$$

Here, the steady-state voltage dependence was chosen to be $h_{L,\infty} = [1 + e^{[(101+V)/6.1]}]^{-1}$. By changing time constant τ_{hL}, we were able to modify the duration of ectopic activity episodes. $\tau_{hL} = 15\,000$ ms was chosen for Fig. 2 and $\tau_{hL} = 3000$ ms for Fig. S5(a) of the Supplemental Material [18].

The forward Euler method was used to integrate the equations with a time step $\Delta t = 0.005$ ms and a centered finite-differencing scheme to discretize the Laplacian with a space step of $\Delta x = 0.0625$ mm. The total computational domain size was 256×256 grid points; the centrally located square with the activated late Na^+ current consisted of 115×115 grid points. To create stationary initial conditions, the model was integrated for 2 min before a single stimulus was applied to the lower border of the domain.

f. Detailed electrophysiological model of adult human ventricular myocytes with regionally activated late Na^+ current

The ten Tusscher-Noble-Noble-Panfilov model was adopted from Ref. [20]. The conductances of the transient outward (G_{to}), rapid (G_{Kr}), and the slow delayed (G_{Ks}) rectifier K^+ current were reduced to 50% of their original values. This did not significantly prolong APD.

The late Na^+ current was implemented in the same manner as for the rat model. We used the same formalism as in Eq. (B4), where the activation kinetics coincides with the kinetics of the fast Na^+ current. The inactivation variable also followed Eq. (B5). The steady-state inactivation was $h_{L,\infty} = [1 + e^{[(101+V)/6.1]}]^{-1}$, where V is the transmembrane potential; the conductance of the channel $G_{\text{NaL}} = 0.65536\ \text{mS}/\mu\text{F}$. Time constants were $\tau_{hL} = 25000$ ms for the prolonged episode in Fig. 2 of the main manuscript and $\tau_{hL} = 5000$ ms for the short episode in Fig. S5(b) of the Supplemental Material [18]. The forward Euler method was used to integrate the equations with a time step $\Delta t = 0.005$ ms and a centered finite-differencing scheme to discretize the Laplacian with a space step $\Delta x = 0.0625$ mm. The diffusion coefficient was equal to $0.000154\ \text{cm}^2/\text{ms}$. The total computational domain size was 1024×1024 grid points; the centrally located square with the activated late Na^+ current consisted of 460×460 grid points. The model was integrated for 2 min to create stationary initial conditions. Thereafter, a single pulse was delivered at the lower border of the domain.

g. Detailed electrophysiological model of adult human ventricular myocytes with regionally increased Ca^{2+} current

The ten Tusscher–Noble–Noble–Panfilov model was adopted from Ref. [20]. The conductance of the transient rapid delayed rectifier (G_{Kr}) was set to zero throughout the computational domain. The time constant of the inactivating f-gate of the L-type Ca^{2+} was reduced twofold uniformly in the domain. The conductance of L-type Ca^{2+} channel G_{CaL} was increased 12 times inside the square to mimic damaged tissue and 2 times in the surrounding tissue, causing a sixfold difference in conductance between the inner and outer regions of the domain. The diffusion coefficient was set

to 0.000154 cm^2/ms. The forward Euler method was used to integrate the equations with $\Delta t = 0.005$ ms. The Laplacian was implemented using a centered finite-differencing scheme with $\Delta x = 0.0625$ mm. The total domain size was 512×512 grid points; the centrally located square with the increased Ca^{2+} current consisted of 230×230 grid points. The model was integrated for 2 min to create stationary initial conditions, after which a stimulus was delivered at the lower domain boundary. The initial wave of excitation led to a sustained depolarization of the square, followed by ectopic waves originating from the corners of the square (Fig. S7 of the Supplemental Material [18]).
